# Prevalence and Risk Factors Associated with Precancerous Cervical Cancer Lesions among HIV-Infected Women in Resource-Limited Settings

**DOI:** 10.1155/2012/953743

**Published:** 2012-04-04

**Authors:** Peter Memiah, Wangeci Mbuthia, Grace Kiiru, Solomon Agbor, Francesca Odhiambo, Sylvia Ojoo, Sibhatu Biadgilign

**Affiliations:** ^1^Institute of Human Virology, University of Maryland School of Medicine, Baltimore, MD, USA; ^2^Nazareth Hospital, Naitobi, Kenya; ^3^Maryland Global Initiatives Corporation Kenya, University of Maryland, College Park, MD, USA; ^4^Department of Epidemiology, College of Public Health and Medical Science, Jimma University, P.O. Box 24414, Addis Ababa, Ethiopia

## Abstract

*Objective*. To assess the prevalence and identified associated risk factors for precancerous cervical cancer lesions among HIV-infected women in resource-limited settings in Kenya. *Methods*. HIV-infected women attending the ART clinic at the Nazareth Hospital ART clinic between June 2009 and September 2010. Multivariate logistic regression model with odds ratios and 95% confidence intervals (CI) were estimated after controlling for important covariates. *Result*. A total of 715 women were screened for cervical cancer. The median age of the participants was 40 years (range 18–69 years). The prevalence of precancerous lesions (CINI, CINII, CIN III, ICC) was 191 (26.7%). After controlling for other variables in logistic regression analysis, cervical precancerous lesions were associated with not being on ART therapy; whereby non-ART were 2.21 times more likely to have precancerous lesions than ART patients [(aOR) = 2.21, 95% CI (1.28–3.83)]. *Conclusion*. The prevalence of precancerous cervical lesions was lower than other similar settings. It is recommended that cancer screening of HIV-infected women should be an established practice. Availability and accessibility of these services can be done through their integration into HIV. Prompt initiation of HAART through an early enrollment into care has an impact on reducing the prevalence and progression of cervical precancerous lesions.

## 1. Background

Cervical cancer is the second most common malignancy and accounts for the greatest number of deaths from cancer in women worldwide [1]. Human immunodeficiency virus (HIV) infection also represents a tremendous health burden worldwide. Cervical cancer was made an acquired immunodeficiency syndrome (AIDS-) defining illness in 1993 [2]. In 2005, there were almost 260,000 deaths from cervical cancer and more than 500,000 new cases worldwide. Women in developing countries are at greater risk of death from cervical cancer primarily because few have access to the screening and treatment services that have greatly reduced mortality in the industrialized world over the past four decades. About 75% of women in industrialized countries has been screened for cervical cancer in the previous five years, compared to less than 5% in developing countries [3]. According to WHO (1986), it has been estimated that only about 5% of women in developing countries has been screened for cervical dysplasia in the past 5 years, compared with 40% to 50% of women in developed countries [4]. Research findings suggest that HIV-induced immunodeficiency predisposes to cervical intraepithelial neoplasia (CIN) or cervical carcinoma by facilitating the expression of a causal agent [5–8]. The public health importance of assessing the interaction between HIV and HPV infection with respect to cervical disease is suggested by increased rates of dysplasia persistence and recurrence among HIV-positive women and shorter survival for women with HIV infection and cervical cancer [9].

HIV-positive women have a higher prevalence and incidence of cervical precancerous lesions than HIV negative women. HIV-positive women have a 2-fold to 4-fold greater rate of HPV infection than HIV-negative women. The prevalence of HPV among HIV-positive women is associated strongly with CD4 counts and HIV viral load (VL) [10, 11]. Highly active antiretroviral therapy (HAART) has been shown to decrease HIV viral loads, increase CD4 cell counts, and decrease most opportunistic infections. Since the introduction of HAART there has been a decline in certain malignancies in HIV-infected individuals [12, 13]. However, studies on the impact of HAART on the natural history of cervical squamous intraepithelial lesions (SILs) have produced inconsistent results [14, 15]. In HIV-infected women, there is an increased risk of HPV infection and squamous intraepithelial lesions (SIL), the precursor of cervical cancer [16, 17]. There is still limited evidence for understanding the natural history and epidemiology of HPV-induced cervical cancer in HIV-infected women. Therefore this study may assist policy makers to develop guidelines for prevention and treatment strategies for cervical cancer among HIV-infected women which is largely based on limited evidence, or in the case of resource limited settings, which currently are completely lacking. The objective of the study was to assess prevalence and associated risk factors for precancerous cervical lesions in HIV-infected women within resource-limited settings in Kenya.

## 2. Methods

The study site was a faith-based hospital offering comprehensive care and treatment to approximately 4,000 HIV-infected patients. The study site was located in Central Kenya, Kiambu district which has a HIV prevalence of 4%. The study population constituted eligible women attending the ART treatment clinics. None of the patients had evidence of Kaposi sarcoma or non-Hodgkin lymphoma. Eligibility criteria included being aged 18–69 years and having no current or past history of cervical disease. Women, eighteen years and older, who were on followup for their HIV-positive status, were screened for cervical cancer using the Visual Inspection with Lugol's Iodine (VILI) technique.

Data on sociodemographic status, sexual behavior, history of a sexually transmitted infection (STI), obstetrics, and gynecology history (parity) was obtained from patient medical records as part of the routine quality improvement activities; CD4 count data and HAART status were also extracted from clinical records. Clients were referred to the clinician where a pelvic examination was conducted using a sterile speculum examination. Visual inspection with lugol's iodine (VILI) was used as the screening technique. A positive VILI test necessitated a cervical biopsy; this was preserved in a sterile container using formalin as the fixative and the biopsies were then taken for histology.

The histology result upon biopsy would turn out to be either negative for intraepithelial lesion (IEL), active/chronic cervicitis, precancerous lesions (CIN I, CIN II, or CIN III/CIS), or cervical cancer (squamous cell or adenocarcinoma) which was either differentiated (well, moderately, poorly differentiated) cervical cancer. The cervical cancer clients were clinically staged using the 1986 International Federation of Gynecology and Obstetrics (FIGO) architectural staging system [18].

Their participation in the screening in no way affected access to, or provision of, comprehensive HIV/AIDS care as this was a standard of care at the clinic.

### 2.1. Statistical Analysis

Statistical analysis was carried out using STATA 11 (StataCorp LP, College Station, TX, USA). Descriptive statistics (medians and proportions) were used to characterize the variables. Bivariate (unadjusted) analysis was performed to identify factors significantly associated with the precancerous lesions. Odds ratios (OR), 95% confidence intervals (CI) and two-tailed *P*  values were calculated. Statistical tests used were the Chi-square and Fischer's exact tests for proportions, median test, and Wilcoxon rank test for continuous variables. Variables found to be statistically significant (*P* < 0.05) on unadjusted analysis were included in a multivariate logistic regression model through a combination of forward and backward stepwise methods.

## 3. Result

### 3.1. Sociodemographic Characteristics of the Study Participants

All the 715 HIV-positive women attending the clinic between June 2009 and December 2010 were included for this study. The median age of the participants was 40 years (range 18–69 years). About 589 (85.5%) were in the reproductive age group (15–49) years. Regarding the parity, 555 (94%) have ≥1 child. About 645 (90.3%) were ever married and 53 (7.4%) were single (Table 1).

### 3.2. Clinical and Reproductive Characteristics

The majority of participants were in WHO stage III 316 (44%) and 210 (29.2%) in WHO stage IV (Table 1). Concerning the baseline CD4 count, 273 (97.5%) ART clients and 7 (2.5%) preART cases were below 200/mm3 while 245 (71.9%) ART and 96 (28.1%) of Pre-aRT cases were above 200/mm3. About 90 (91.8%) Pre-ART and 8 (8.2%) ART of the study cases were below 200 in their current CD4 count and 488 (82.9%) ART and 100 (17.1%) Pre-ART cases were ≥200 in their current CD4 (Table 2). The median and mean baseline CD4 cell count were 228.0/mm3 and 251/mm3 (range 1–1547.0/mm3). The median and mean current CD4 cell count were 438.0/mm3 and 458/mm3 (range 5–1547.0/mm3). About 578 (84.3%) women with HIV infection were on antiretroviral therapy (ART). About 642 (92.6%) of the HIV-infected women were in followup period of ≥1 year. About 610 (85.4%) of the participants did not have any history of abortion and 104 (14.6%) had history of abortion at least once in life time. The prevalence of precancerous lesions (CIN I, CIN II, CIN III, and ICC) was 191 (26.7%) (Table 1).

### 3.3. Factors Associated with Precancerous Cervical Cancer Lesions in HIV-Infected Women

In the bivariate analysis (Table 3), only two variables are found to be associated with precancerous lesions. Those women whose current CD4 count was less than 200 were 1.6 times more likely to have precancerous lesions than those patients above 200 current CD4 count [crude odds ratios (cOR) = 1.61, 95% CI (1.02–2.53)]. Non-ART patients were 2.2 times more likely to have precancerous cervical lesions than those patients on ART [crude odds ratios (cOR) = 2.23, 95% CI (1.29–3.86)]. In multivariate analysis after controlling other variables, only non-ART patients were 2.21 times more likely to have precancerous lesions than patients on ART patient [adjusted odds ratios (aOR) = 2.21, 95% CI (1.28–3.83)].

## 4. Discussion

According to the World Health Organization (WHO), invasive cervical cancer (ICC) is the second most common cancer in women worldwide and is more frequent in low-income countries [19]. Recent guidelines recommend that, following two initial normal Pap-smears at a 6-month interval, all HIV-positive women should undergo annual cervical cytologic examination. In addition, it is recommended that all immunosuppressed women with atypical squamous cells undergo colposcopy [20]. In our study the prevalence of precancerous lesions was 26.7% (191). In other studies among HIV-infected women in Lusaka, Zambia was 79% prevalence of high-grade squamous intraepithelial lesions (HSIL) of the cervix [21] and the high prevalence of HPV-DNA in Zambia study (97.2% for any HPV and 90.3% for any HR-HPV) [22]. In Guinea, overall human papillomavirus prevalence was 50.8% (78.5% and 47.9% among women with and without cervical abnormalities, resp.) [23]. A recent meta-analysis shows that the HPV prevalence was 56.6% in Africa, 31.1% in Asia, 32.4% in Europe, 31.4% in North America, and 57.3% in South/Central America [24]. Although the mechanism by which HIV increases risk of cervical cancer is not completely understood, studies suggest that HIV-induced immunosuppression leads to an inability to control the expression of HPV and the production of HPV oncoproteins E6 and E7 [25, 26] and the risk appears to be associated with increased HPV persistence that may result from immunosuppression related to HIV. Furthermore, the risk is greater in women with CD4 counts less than 200 cells per microliter and in those with high plasma HIV RNA levels [27]. Studies have shown that HIV-1 infection is associated with an increased rate of HPV infection, mainly restricted to HR-HPV types which are the cause of invasive cancer of the cervix [28–30]. Several studies conducted in sub-Saharan Africa indicate that among African women, being HIV infected was associated with a high risk of presenting squamous intraepithelial lesions of the cervix, with ORs ranging from 4.4 to 17 depending on the grade of the lesion and other factors [28, 30–33]. Yet, many case-referent studies conducted in Uganda, Rwanda, and Côte d'Ivoire failed to demonstrate any significant association between HIV and ICC, with ORs ranging from 1.1 to 1.6 [34–37]. Case-referent studies in South Africa also found a significant association with ORs of 1.6 and 1.6 [38, 39]. In another case-referent study conducted in Kenya, HIV-positive women were also more likely to be HPV infected compared to HIV-negative women (OR = 3.1) [40]. A cross-sectional study determined the prevalence of HPV infection, HIV infection, and cervical cytological abnormalities in Ugandan women presenting to a sexually transmitted infection clinic [41] and found 49 cases of HPV infection among 106 women with cervical swabs adequate for HPV testing (46.2%). Similarly, a study realized in Zambia among 145 HIV-infected women of which 93.8% had abnormal Pap smears [22]. In other study of women initiating ARV therapy recorded an exceptionally high prevalence of cervical abnormalities (66%) [42]. This finding has implications for future interventions in the implementation of prophylactic HPV vaccines to be the part of care and support programme for HIV-infected women. 

In our study those women whose current CD4 count was less than 200 were 1.6 times more likely to have precancerous cervical cancer lesions than those patients with a current CD4 count above 200. However, regarding the effect of immune function, previous studies have consistently demonstrated that the amount of CD4 cell count is a significant predictor for having or developing CIN [43–45]. Given the low CD4 counts in our study population, severe immunosuppression is the most likely explanation for the high prevalence of cervical abnormalities and precancerous cervical lesions detected.

In HIV infection, lower CD4 counts have been associated with a higher prevalence of HPV infection [5, 46] and persistent shedding of HPV DNA [47, 48]. HPV viral load increases with immune suppression, likely accounting for greater facility of HPV DNA detection [46]. In this study there was a positive association between having a CD4 count of <200. This suggests that as the CD4 count declines, vigilant followup of the anogenital tract, particularly with cervical cytological and/or histological screening, is warranted [49].

In our analysis non-ART patients were 2.21 times more likely to have CIN infection than ART patients. Similar findings have been documented in other studies. As HIV-infected women continue to live longer with ART support, albeit in a moderately immunosuppressed state, they may be at increased risk for CIN and invasive cervical cancer [11, 50]. However, studies on the impact of HAART on the natural history of cervical squamous intraepithelial lesions have produced inconsistent results [14, 15]. Antiretroviral therapy (ART) for PLHIV provides a golden opportunity to increase cervical screening through the integration of ART services with frequent screening of women for cervical cancer [21, 50]. Starting HAART has an impact on reducing the incidence and progression and facilitating the regression of CIN infection and cervical abnormalities.

The study should be interpreted in light of the strength and limitations. Major strengths of our study include the large number of women, high participation, and reliance on cost-effective, simple, timely VILI testing. The Limitations include but not limited to the lack of information on some of the variables that can predict cervical cancer in HIV-infected women like the use of oral contraceptives or estrogens, information on sexual behavior, and smoking [49] and the cross-sectional nature of this study allows the possibility for confounding so it is difficult to reach any causality. This may hence hinder the generalizability of our findings.

## 5. Conclusion

In this study, the prevalence of CIN was 26.7 percent (191) which is lower than other findings reported in Africa. Those patients who were not on antiretroviral therapy were more likely to have CIN diagnosis than who were on antiretroviral therapy. Therefore regular screening of HIV-infected women is of paramount importance. Starting ART acts as leverage for cancer screening and majority of ART patients are likely not to progress to cervical cancer as demonstrated by this study. Conducting well-designed prospective cohort study to study natural history of cervical neoplasia among HIV-infected women in developing country settings is warranted.

## Figures and Tables

**Table 1:** Sociodemographic, clinical, and reproductive characteristics of HIV+ women screened for cervical cancer at Nazareth Hospital.

Variables	Sample size (*n*)	Percentage (%)	Mean (SD)
HAART status (*n* = 686)			
Yes	108	15.7	—
No	578	84.3	—

Presence of precancerous lesions (*n* = 715)			
Negative	524	73.3	—
Positive	191	26.7	—

Marital status (*n* = 714)			
Deceased	10	1.4	—
Married	645	90.3	—
Separated	6	0.8	—
Single	53	7.5	—

Age (*n* = 689)			40 (8.72)
	57	8.27	—
30–39	302	43.83	—
40–49	230	33.38	—
50–59	77	11.18	—
60–69	23	3.34	—

Baseline CD4 (*n* = 621)			250 (189)
<200	280	45.0	—
	341	55.0	—

Current CD4 (*n* = 686)			458 (236)
	98	14.2	—
	588	85.8	—

WHO staging (*n* = 692)			
Stage 1	80	11.6	—
Stage 2	88	12.7	—
Stage 3	316	45.7	—
Stage 4	208	30.0	—

History of abortion (*n* = 714)			
None	610	85.4	—
≥1	104	14.6	—

Parity (*n* = 591)			2.81 (1.95)
0	36	6.0	—
1-2	264	44.6	—
	291	49.4	—

Duration of followup in care (*n* = 693)			2.62 (1.54)
	51	7.4	—
1–3 years	443	63.9	—
4–6 years	199	28.7	—

Histology results (*n* = 191)			
Normal	112	58.6	
CIN 1	35	18.3	
CIN 2	20	10.5	
CIN 3	17	8.9	
ICC	7	3.7	

Note: SD stands for standard deviation.

**Table 2:** CD4 and ART characteristics of HIV+ women screened for cervical cancer in Nazareth Hospital.

CD4 count	on ART	
	No	Yes
Baseline CD4 count (*n* = 568)		
<200	7 (2.5)	273 (97.5)
≥200	96 (28.1)	245 (71.9)
Current CD4 count (*n* = 686)		
<200	8 (8.2)	90 (91.8)
≥200	100 (17.1)	488 (82.9)

**Table 3:** Unadjusted and adjusted odds ratios of the multilevel logistic regression estimates for factors associated with precancerous cervical cancer lesions among HIV+ at Nazareth Hospital.

Characteristics	Cervical cancer screening		*P* value	Unadjusted/cOR	Adjusted/aOR	*P* value
	Negative	Positive				
Current CD4 count			*0.038*			
<200	78 (72.2)	30 (27.8)		1.61 (1.02–2.53)		
≥200	431 (87.5)	147 (81.3)		1.00		
On HAART			*0.003 *			*0.005*
Yes	91 (84.2)	17 (15.8)		1.00	1.00	
No	404 (69.8)	174 (30.2)		2.23 (1.29–3.86)	2.21 (1.28–3.83)	
